# L-Cysteine Ethyl Ester May Overcome Morphine-Induced Respiratory Depression by Activating Muscarinic Receptors

**DOI:** 10.3390/ph19071125

**Published:** 2026-07-21

**Authors:** Paulina M. Getsy, Walter J. May, Santhosh M. Baby, Gregory A. Coffee, Hubert V. Forster, Matthew R. Hodges, Yunguang Qiu, Feixiong Cheng, James N. Bates, Stephen J. Lewis

**Affiliations:** 1 Department of Pediatrics, Case Western Reserve University, Biomedical Research Building, Room 831, 10900 Euclid Avenue, Cleveland, OH 44106, USA; pxg55@case.edu (P.M.G.); gac43@case.edu (G.A.C.); 2 Department of Pediatrics, University of Virginia, Charlottesville, VA 22903, USA; walterjmay@gmail.com; 3 Galleon Pharmaceuticals, Inc., 213 Witmer Road, Horsham, PA 19044, USA; babysanthosh@gmail.com; 4 Department of Physiology, Medical College of Wisconsin, Milwaukee, WI 53226, USA; bforster@mcw.edu (H.V.F.); mhodges@mcw.edu (M.R.H.); 5 Zablocki Veterans Affairs Medical Center, Milwaukee, WI 53295, USA; 6 Neuroscience Research Center, Medical College of Wisconsin, Milwaukee, WI 53226, USA; 7 Genomic Medicine Institute, Lerner Research Institute, Cleveland Clinic, Cleveland, OH 44195, USA; qiuy3@ccf.org (Y.Q.); chengf@ccf.org (F.C.); 8 Department of Molecular Medicine, Cleveland Clinic Lerner College of Medicine, Case Western Reserve University, Cleveland, OH 44195, USA; 9 Case Comprehensive Cancer Center, School of Medicine, Case Western Reserve University, Cleveland, OH 44106, USA; 10 Department of Anesthesiology, University of Iowa Hospitals and Clinics, Iowa City, IA 52242, USA; jnbates25@gmail.com; 11 Department of Pharmacology, Case Western Reserve University, Cleveland, OH 44106, USA

**Keywords:** morphine, atropine, L-cysteine ethyl ester, muscarinic cholinergic receptors, hypoxic-hypercapnic gas challenge, ventilatory parameters, rats

## Abstract

**Background/Objectives:** Opioids inhibit breathing that can lead to fatal overdose, highlighting the need for testing effective countermeasure agents and potential mechanisms of action. Here we examined the role muscarinic cholinergic receptors play in the ability of L-cysteine ethyl ester (L-CYSee) to overcome the deleterious effects of morphine on ventilatory parameters in male Sprague Dawley rats and the ventilatory responses during a subsequent hypoxic-hypercapnic (HH) challenge. **Methods:** Ventilatory parameters were measured by whole body plethysmography. **Results:** The injection of the muscarinic receptor antagonist, atropine (1.0 mg/kg, IV), elicited an array of ventilatory responses (e.g., an increase in frequency of breathing coupled with a fall in tidal volume). Injection of morphine (10 mg/kg, IV) to vehicle-treated rats elicited a depression of breathing, including sustained decreases in tidal volume, minute ventilation, peak inspiratory flow, and peak inspiratory and expiratory drives, which were associated with marked increases in end inspiratory pause (EIP) and end expiratory pause (EEP), expiratory flow at 50% expired tidal volume (EF_50_), and rate of achieving peak expiratory flow (Rpef). Most effects of morphine (10 mg/kg, IV) were not altered in atropine-treated rats, except that increases in EIP, EEP and Rpef were reduced. Subsequent injections of L-CYSee (2 × 500 μmol/kg, IV given 15 min apart) overcame the adverse actions of morphine on ventilatory parameters in vehicle-treated rats. The effects of L-CYSee, such as reversal of the effects of morphine on frequency of breathing, tidal volume and minute ventilation, were markedly reduced in atropine-treated rats. The ability of L-CYSee to reverse the adverse effects of morphine to a HH gas challenge was markedly diminished in atropine-treated rats. **Conclusions:** These findings demonstrate that muscarinic receptors play a vital role in the ability of L-CYSee to overcome the deleterious effects of morphine.

## 1. Introduction

The adverse effects of opioids on breathing limit their usefulness as analgesics [1,2,3]. Opioid receptor antagonists such as naloxone (Narcan^®^) can overcome opioid-induced respiratory depression (OIRD) but they block analgesia and often elicit life-threatening withdrawal in opioid-dependent subjects [1,2,3]. Several drug classes without opioid receptor antagonist efficacy have been tested for efficacy against OIRD although none have attained clinical efficacy (see Table S1 in Getsy et al. [4]). Developing a unifying hypothesis as to how opioids cause OIRD would drive the development of drugs that reverse OIRD while preserving analgesia. Trivedi et al. [5] established that morphine elicits cell-signaling cascades that block excitatory amino acid transporter type 3 (EAA3)-mediated active transport of L-cysteine into neurons. Trivedi and Deth [6] proposed that the resulting changes in redox status of neurons and glia to a more oxidative state as well as decreased participation of L-cysteine in cell-signaling pathways, drive the development of addiction to opioids. The work of Trivedi and colleagues [5,6] and others [7,8,9,10,11,12] led us to consider whether a loss of L-cysteine entry into cells may be involved in the expression of OIRD and whether membrane-permeable analogues of L-cysteine may overcome OIRD. We tested cell-permeable ethyl esters and methyl esters of L-cysteine (ester linkage to carboxyl moiety) and found that L-cysteine ethyl ester (L-CYSee) and L-cysteine methyl ester (L-CYSme) overcome the adverse effects of morphine on ventilatory parameters, alveolar gas-exchange and arterial blood-gas chemistry (pH, pCO_2_, pO_2_, sO_2_) in male Sprague-Dawley rats while preserving morphine analgesia [13,14].

It is not clear whether L-CYSee to overcomes OIRD by its redox effects or whether it elicits cell-signaling cascades via the activation of receptors, ion channels, and/or enzymes. The inability of potent redox regulators such as N-acetyl-L-cysteine methyl ester (L-NACme) and L-serine ethyl ester (L-SERee, oxygen atom instead of cysteine atom as in L-CYSee) to overcome OIRD elicited by bolus injections of opioids [13,14] suggests a two-step process by which L-CYSee overcomes OIRD, namely (1) restoration of the redox status of cells thereby restoring proper function of all signaling cascades (including opioid receptor signaling), and (2) L-CYSee binding to signaling elements (e.g., plasma membrane receptors) that L-NACme or L-SERee do not. Cholinergic receptors (nicotinic and muscarinic) within the brain [15,16,17,18,19] and the carotid bodies [20,21,22,23] play complex roles in the control of breathing and the processing of chemoreceptor input from the carotid bodies. Moreover, acetylcholinesterase inhibitors [24,25,26] and nicotinic receptor agonists [27,28] overcome some of the adverse effects of opioids. To date, the potential roles of central or peripheral muscarinic receptors in the expression of OIRD in freely moving rats have not been explored. However, our companion manuscript [29] reports that the effects of morphine on some ventilatory parameters were overcome by atropine, a peripheral/central-acting M1–M5 muscarinic receptor antagonist [30]. Distribution/expression levels of M1-M5 receptors differ in central ventilatory control centers [31,32,33,34] and carotid body structures, including primary glomus cells [35,36,37,38,39], as do the signal transduction processes activated by these receptors [30,31,32,33,34,40]. M1, M3, and M5 receptors are Gq/11 protein-linked receptors found mostly post-synaptically, and their stimulation initiates phospholipase C-mediated increases in intracellular Ca^2+^ [31,32,33,34,40]. M2 and M4 receptors are Go/i protein-coupled receptors that exist pre- and post-synaptically and cause a decrease in intracellular cAMP via inhibition of adenylyl cyclase [40].

The use of whole-body plethysmography allows for detailed analyses of the effects of drugs such as morphine and atropine and manipulations such as hypoxic-hypercapnic (H-H) challenges on ventilatory parameters in unanesthetized freely moving (unrestrained) rats [4,13,14]. This plethysmography system provides real-time data pertaining to the temporal changes in ventilatory parameters during the designated manipulations within a particular protocol, allowing, in the present instance, analyses of the efficacy of atropine in modulating the ability of L-CYSee to overcome the actions of morphine on ventilatory parameters.

The major objectives of the present study were to determine the role of muscarinic receptors in L-CYSee’s ability to overcome the deleterious effects of morphine on ventilatory parameters and the responses elicited by H-H challenge [41]. One group of rats received a standard dose (1.0 mg/kg, IV) of atropine [42,43,44] followed by an injection of morphine (10 mg/kg, IV) and then two injections of L-CYSee (500 μmol/kg, IV) with all injections given 15 min apart. Thirty min after the second injection of L-CYSee, the rats received a rebreathing HH-challenge of 60 min followed by return to room air for 30 min. A second group of rats underwent the same protocol but received an injection of vehicle rather than atropine. Our findings suggest that muscarinic receptors play a vital role in the ability of L-CYSee to overcome the adverse effects of morphine on ventilatory parameters *per se* and the ventilatory responses elicited during and following H-H challenge.

## 2. Results

### 2.1. Baseline Parameters

The ages, body weights and ventilatory values recorded prior to injection of vehicle (saline) in one group of rats or atropine in another group are provided in Table S2. There were no between-group differences for any parameter (*p* > 0.05, for all comparisons). Note that definitions of the ventilatory parameters to be discussed below are provided in Table S1.

### 2.2. Ventilatory Studies

#### 2.2.1. Frequency of Breathing (Freq), Tidal Volume (TV) and Minute Ventilation (MV)

**Figure 1:**
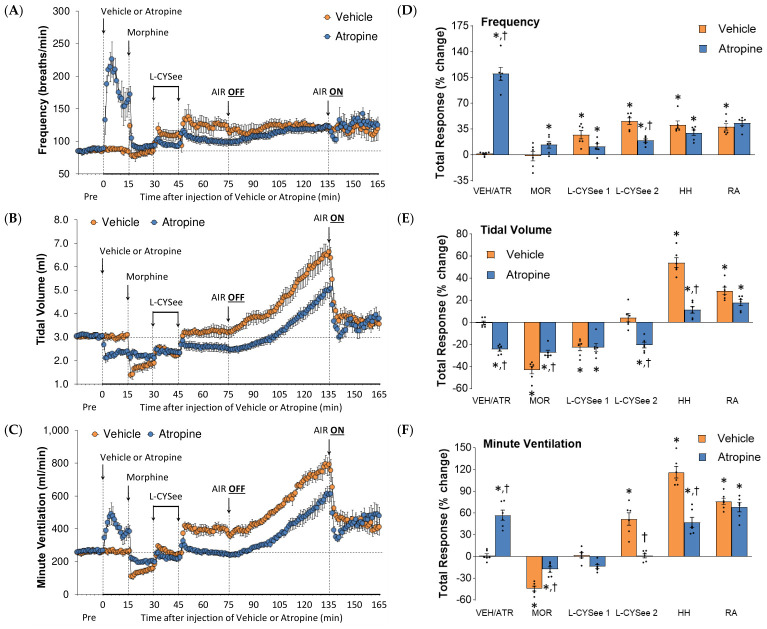
Frequency of breathing (Panel (**A**)), tidal volume (Panel (**B**)), and minute ventilation (Panel (**C**)) before (Pre), after injection of vehicle (VEH) or atropine (1 mg/kg, IV), then injection of morphine (10 mg/kg, IV), and finally subsequent injections of L-cysteine ethyl ester (L-CYSee, 2 × 500 μmol/kg, IV) followed by a hypoxic-hypercapnic gas challenge (AIR OFF) and return to room-air (AIR ON). A summary of the total changes in frequency, tidal volume, and minute ventilation during each phase of the study is presented in Panels (**D**–**F**), respectively (ATR—atropine; HH—hypoxic-hypercapnic gas challenge; RA—return to room-air). The data are presented as mean ± SEM. There were six rats in each group. * *p* < 0.05, significant change from Pre-values. ^†^ *p* < 0.05, atropine versus vehicle.

This section describes our findings regarding the frequency of breathing (Freq), the depth of breathing (tidal volume, TV), and the total volume of air breathed in per minute (Freq × TV = minute ventilation, MV). As seen in Figure 1, the injection of atropine (1.0 mg/kg, IV) elicited a pronounced and sustained increase in Freq (Panel A) but a sustained decrease in TV (Panel B) that together caused a sustained increase in MV (Panel C). The vehicle injection elicited minor responses. Subsequent injection of morphine elicited a minor fall in Freq, accompanied by a pronounced and sustained decrease in TV in vehicle-treated rats that together produced a sustained decrease in MV. The injection of morphine (10 mg/kg, IV) elicited a prompt reversal of the atropine-induced increase in Freq, with values returning to pre-atropine levels. Morphine did not further lower TV in atropine-treated rats, such that the fall in MV was smaller in atropine-treated rats than in vehicle-treated rats.

The two injections of L-CYSee (500 μmol/kg, IV) produced substantial increases in Freq, TV and MV in vehicle-treated rats. The responses elicited by the injections of L-CYSee (and in particular, injection 2) were markedly smaller in atropine-treated rats. The subsequent HH-challenge did not alter Freq in vehicle-treated rats, whereas it elicited a pronounced increase in TV and, consequently, MV. The peak increases in TV and MV were markedly smaller in atropine-treated rats, noting that the temporal responses tracked in parallel fashion. The return to room air responses (recovery toward baselines) were similar in both groups. A summary of the total changes in Freq, TV and MV during each phase of the protocol is shown in Panels D–F, respectively (VEH—vehicle; ATR—atropine; MOR—morphine; HH—hypoxic-hypercapnic challenge; RA—return to room air). These summaries confirm that (a) atropine caused a pronounced increase in Freq accompanied by a less pronounced decrease in TV, resulting in an increase in MV, and (b) atropine markedly blunts the responses elicited by the second injection of L-CYSee, with the HH-challenge-induced changes in TV and MV being substantially blunted by atropine.

#### 2.2.2. Inspiratory Time (Ti), Expiratory Time (Te) and Ti/Te

**Figure 2:**
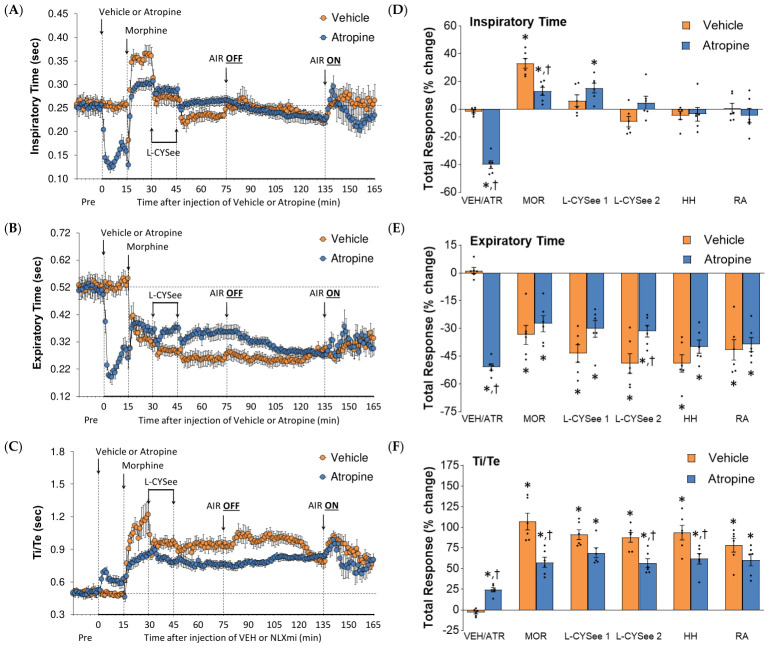
Inspiratory time (Ti) (Panel (**A**)), expiratory time (Te) (Panel (**B**)), and inspiratory time/expiratory time (Ti/Te) (Panel (**C**)) before (Pre), after injection of vehicle (VEH) or atropine (1 mg/kg, IV), then injection of morphine (10 mg/kg, IV), and finally subsequent injections of L-cysteine ethyl ester (L-CYSee, 2 × 500 μmol/kg, IV) followed by a hypoxic-hypercapnic gas challenge (AIR OFF) and return to room-air (AIR ON). A summary of the total changes in inspiratory time, expiratory time, and Ti/Te during each phase of the study is presented in Panels (**D**–**F**), respectively (ATR—atropine; HH—hypoxic-hypercapnic gas challenge; RA—return to room-air). Data are presented as mean ± SEM. There were six rats in each group. * *p* < 0.05, significant change from Pre-values. ^†^ *p* < 0.05, atropine versus vehicle.

This section describes our findings regarding the duration of inspiration (inspiratory time, Ti), the duration of expiration (expiratory time, Te) and inspiratory quotient Ti/Te). As seen in Figure 2, the injection of atropine elicited pronounced and sustained decreases in Ti (Panel A) and Te (Panel B) that together increased Ti/Te (Panel C). Morphine elicited a substantial increase in Ti in vehicle-treated rats, accompanied by a greater fall in Te such that Ti/Te rose. The injection of morphine elicited smaller peak increases in Ti and slightly less pronounced decreases in Te in atropine-treated rats than in saline-treated rats, such that Ti/Te rose less in atropine-treated rats. Atropine diminished the ability of L-CYSee to lower Ti and Te and associated increase in Ti/Te. The HH-challenge elicited minimal changes in Ti, Te, and Ti/Te in saline-treated rats, and these values in both groups reached similar levels by the end of the HH-challenge. The return-to-room-air responses were similar in both groups of rats. As summarized in Panels D–F, atropine elicited substantial decreases in Ti and Te, summing to an increase in Ti/Te. The summary data confirm that the changes in Ti and Te elicited by L-CYSee injections were markedly attenuated by atropine.

#### 2.2.3. End Inspiratory Pause (EIP) and End Expiratory Pause (EEP)

**Figure 3:**
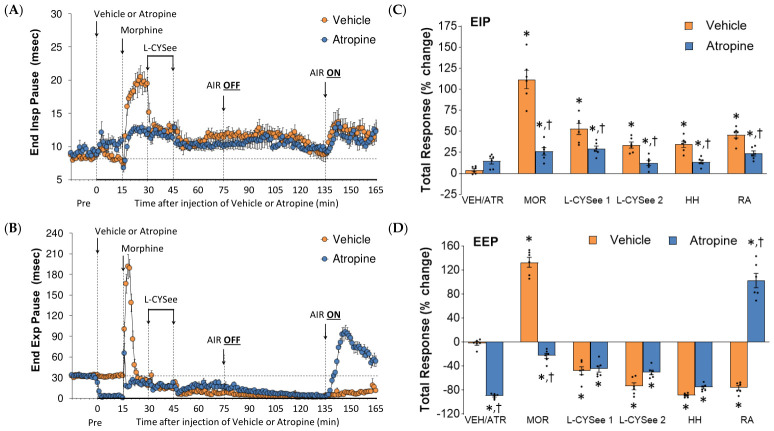
Values recorded for end inspiratory pause (End Insp Pause, EIP) (Panel (**A**)) and end expiratory pause (End Exp Pause, EEP) (Panel (**B**)) before (Pre), after injection of vehicle (VEH) or atropine (1 mg/kg, IV), then injection of morphine (10 mg/kg, IV), and finally subsequent injections of L-cysteine ethyl ester (L-CYSee, 2 × 500 μmol/kg, IV) followed by a hypoxic-hypercapnic gas challenge (AIR OFF) and return to room-air (AIR ON). A summary of the total changes in end inspiratory pause and end expiratory pause during each phase of the study is shown in (Panels (**C**,**D**)), respectively (ATR—atropine; HH—hypoxic-hypercapnic gas challenge; RA—return to room-air). The data are presented as mean ± SEM. There were six rats in each group. * *p* < 0.05, significant change from Pre-values. ^†^ *p* < 0.05, atropine versus vehicle.

This section describes our findings regarding the pause between the end of inspiration and the start of expiration (end-inspiratory pause, EIP), and the pause between the end of expiration and the start of inspiration (end-expiratory pause, EIP). As seen in Figure 3, atropine elicited minor increases in EIP (Panel A) but a profound fall in EEP (Panel B). The morphine-induced increases in EIP and EEP seen in vehicle-treated rats were markedly reduced in the atropine-treated rats. Each dose of L-CYSee had minor effects on EIP and EEP, with the effects on EIP being slightly lower in the atropine-treated rats. There were minor between-group differences during the HH-challenge. A major observation was that the return to room air elicited a substantial and sustained increase in EEP in atropine-treated rats. As summarized in Panels D and E, atropine elicited substantial falls in EEP, with the key finding being the increase in EEP upon return to room air in atropine-treated rats.

#### 2.2.4. Peak Inspiratory Flow (PIF), Peak Expiratory Flow (PEF) and PIF/PEF

**Figure 4:**
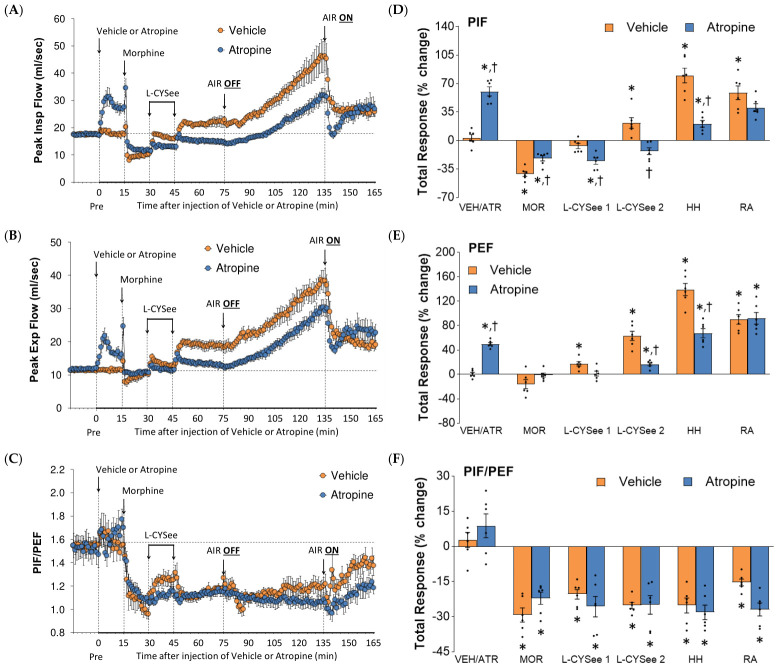
Values recorded for peak inspiratory flow (Peak Insp Flow, PIF) (Panel (**A**)), peak expiratory flow (Peak Exp Flow, PEF) (Panel (**B**)), and peak inspiratory flow/peak expiratory flow (PIF/PEF) (Panel (**C**)) before (Pre), after injection of vehicle (VEH) or atropine (1 mg/kg, IV), then injection of morphine (10 mg/kg, IV), and finally subsequent injections of L-cysteine ethyl ester (L-CYSee, 2 × 500 μmol/kg, IV) followed by a hypoxic-hypercapnic gas challenge (AIR OFF) and return to room-air (AIR ON). A summary of the total changes in PIF, PEF and PIF/PEF during each phase of the study is presented in Panels (**D**–**F**), respectively (ATR—atropine; HH—hypoxic-hypercapnic gas challenge; RA—return to room-air). The data are presented as mean ± SEM. There were six rats in each group. * *p* < 0.05, significant change from Pre-values. ^†^ *p* < 0.05, atropine versus vehicle.

This section describes our findings regarding the peak values attained for inspiratory flows (PIF) and expiratory flows (PEF), and the balance (ratio) of these flows (PIF/PEF). As seen in Figure 4, atropine elicited pronounced and sustained increases in PIF (Panel A) and PEF (Panel B) that together produced minimal changes in PIF/PEF (Panel C). The subsequent injection of morphine elicited a more profound decrease in PIF than PEF in saline-treated rats, resulting in a decrease in PIF/PEF. The ability of L-CYSee to increase PIF and PEF was reduced in atropine-treated rats. The increases in PIF and PEF elicited by a subsequent HH-challenge occurred in parallel in both groups, and return to room air responses were minimally different between the groups. As seen in Panels D–F, atropine elicited substantial increases in PIF and PEF, with the major finding being that the L-CYSee-induced increases in PIF and PEF were markedly reduced in atropine-treated rats.

#### 2.2.5. Expiratory Flow at 50% Expired Tidal Volume (EF_50_) and Rate of Achieving PEF (Rpef)

**Figure 5:**
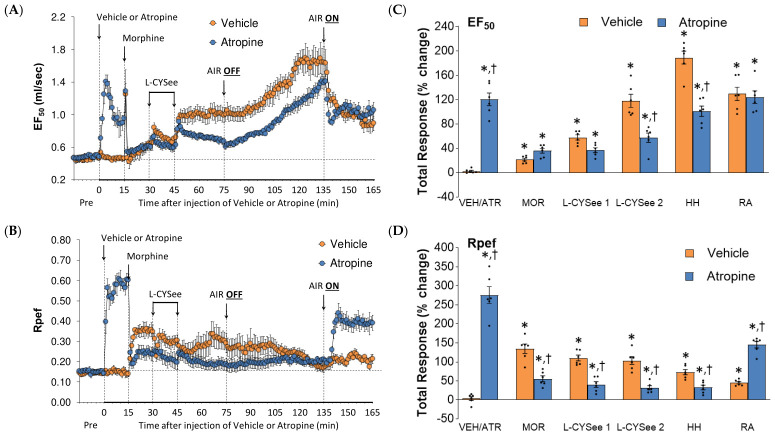
Values recorded for expiratory flow at 50% expired tidal volume (EF_50_) (Panel (**A**)) and rate of achieving peak expiratory flow (Rpef) (Panel (**B**)) before (Pre), after injection of vehicle (VEH) or atropine (1 mg/kg, IV), then injection of morphine (10 mg/kg, IV), and finally subsequent injections of L-cysteine ethyl ester (L-CYSee, 2 × 500 μmol/kg, IV) followed by a hypoxic-hypercapnic gas challenge (AIR OFF) and return to room-air (AIR ON). A summary of the total changes in EF_50_ and Rpef during each phase of the study is presented in (Panels (**C**,**D**)), respectively (ATR—atropine; HH—hypoxic-hypercapnic gas challenge; RA—return to room-air). The data are presented as mean ± SEM. There were six rats in each group. * *p* < 0.05, significant change from Pre-values. ^†^ *p* < 0.05, atropine versus vehicle.

This section describes our findings regarding the peak expiratory flow at 50% expired tidal volume (EF_50_) and the rate of achieving peak expiratory flow (Rpef). As seen in Figure 5, atropine elicited substantial and sustained increases in EF_50_ (Panel A) and Rpef (Panel B). Morphine elicited profound falls in EF_50_ and Rpef, with plateau levels of Rpef in atropine-treated rats being below those in vehicle-treated rats. The L-CYSee-induced increases in EF_50_ and Rpef were markedly reduced in atropine-treated rats. The increases in EF_50_ elicited by HH-challenge occurred in parallel in the two groups, whereas the increases in Rpef were minimal in the atropine-treated rats. A major observation was that the return to room air elicited a substantial and sustained increase in Rpef in atropine-treated rats. As summarized in Panels D and E, atropine elicited substantial increases in EF_50_ and Rpef, with the major observations being that the L-CYSee-induced increases in these parameters were markedly reduced by atropine, whereas there was a marked increase in Rpef upon return to room air in atropine-treated rats.

#### 2.2.6. Relaxation Time (RT), Expiratory Delay (Te-RT) and Apneic Pause [(RT/Te)-1]

**Figure 6:**
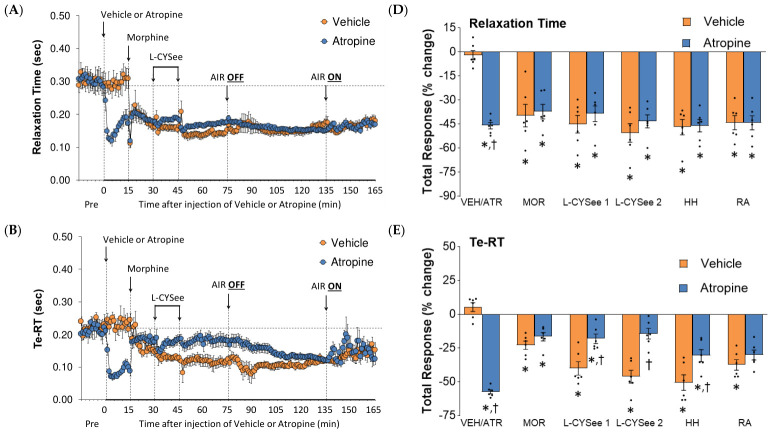

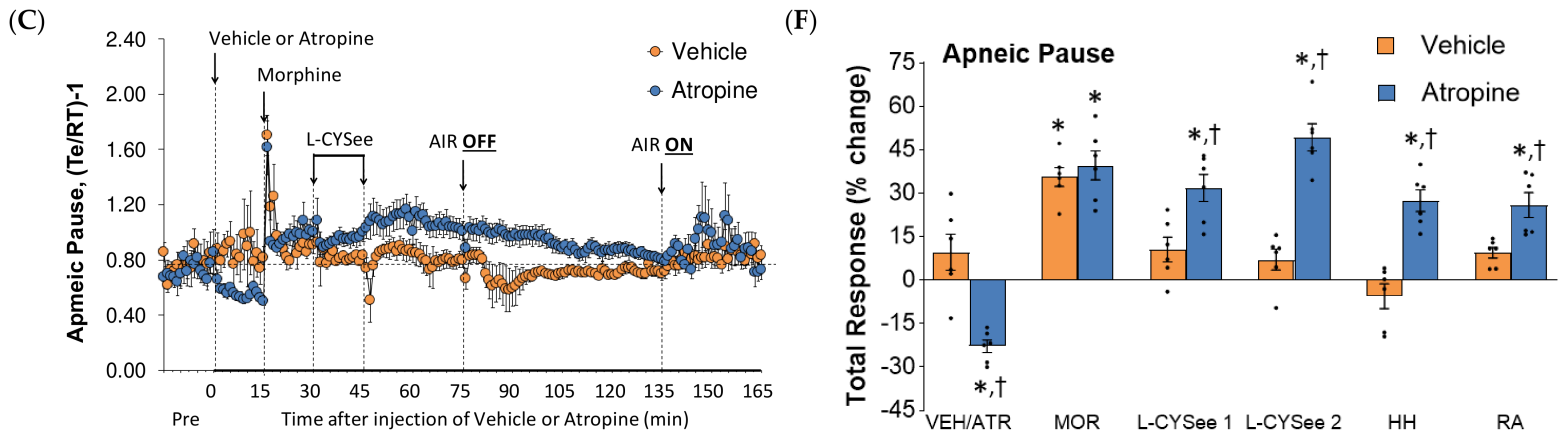
Values recorded for relaxation time (RT) (Panel (**A**)), expiratory time-relaxation time (Te-RT) (Panel (**B**)), and apneic pause ((Te/RT)-1) (Panel (**C**)) before (Pre), after injection of vehicle (VEH) or atropine (1 mg/kg, IV), then morphine (10 mg/kg, IV), and finally subsequent injections of L-cysteine ethyl ester (L-CYSee, 2 × 500 μmol/kg, IV) followed by a hypoxic-hypercapnic gas challenge (AIR OFF) and return to room-air (AIR ON). A summary of the total changes in relaxation time, Te-RT, and apneic pause during each phase of the study is shown in Panels (**D**–**F**), respectively (ATR—atropine; HH—hypoxic-hypercapnic gas challenge; RA—return to room-air). The data are presented as mean ± SEM. There were six rats in each group. * *p* < 0.05, significant change from Pre-values. ^†^ *p* < 0.05, atropine versus vehicle.

This section describes our findings regarding changes in relaxation time (RT) defined as the time taken for expiration to decay to 36% maximum; expiratory delay (Te-RT) which is the difference in duration of expiratory time and relaxation time, and apneic pause (AP), defined as the fraction that relaxation time is of expiratory time [(Te/RT)-1]. As seen in Figure 6, atropine elicited pronounced and sustained decreases in RT (Panel A), expiratory delay (Panel B) and apneic pause (Panel C). Morphine elicited a pronounced decrease in RT and expiratory delay but minimal changes in apneic pause in vehicle-treated rats. Plateau levels of these parameters were similar in atropine-treated rats. The injections of L-CYSee elicited minor responses in both groups, although resting levels of expiratory delay and apneic pause stayed higher in atropine-treated rats. RT, expiratory delay and apneic pause reached similar values by the end of the HH-challenge in both groups, and the return to room air caused minimal responses in both groups. As summarized in Panels D–F, atropine elicited substantial decreases in all three parameters, with major observations being that L-CYSee-induced changes in expiratory delay and apneic pause, but not RT were markedly altered by atropine.

#### 2.2.7. Inspiratory Drive (InspD) and Expiratory Drive (ExpD)

**Figure 7:**
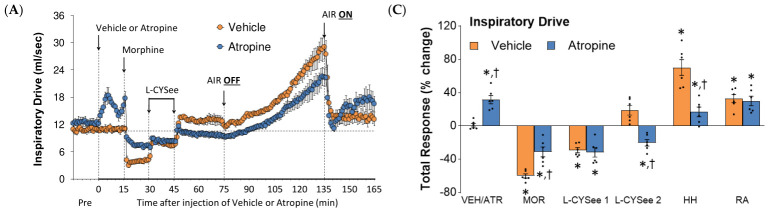

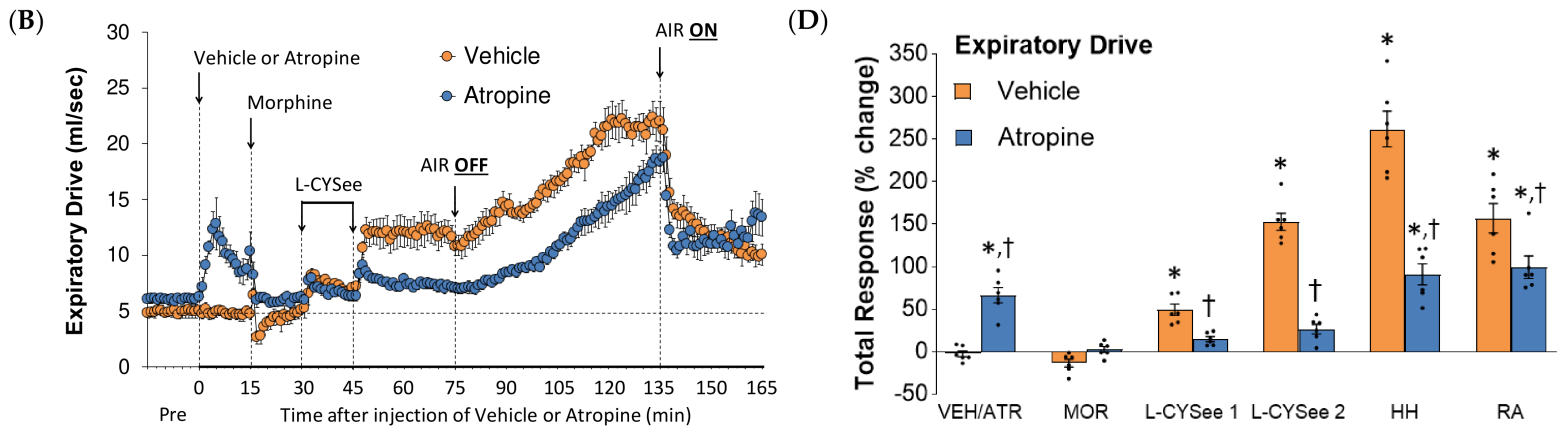
Values recorded for inspiratory drive (Panel (**A**)) and expiratory drive (Panel (**B**)) before (Pre), after injection of vehicle (VEH) or atropine (1 mg/kg, IV), then injection of morphine (10 mg/kg, IV), and finally subsequent injections of L-cysteine ethyl ester (L-CYSee, 2 × 500 μmol/kg, IV) followed by a hypoxic-hypercapnic gas challenge (AIR OFF) and return to room-air (AIR ON). A summary of the total changes in inspiratory drive and expiratory drive during each phase of the study is presented in (Panels (**C**,**D**)), respectively (ATR—atropine; HH—hypoxic-hypercapnic gas challenge; RA—return to room-air). The data are presented as mean ± SEM. There were six rats in each group. * *p* < 0.05, significant change from Pre-values. ^†^ *p* < 0.05, atropine versus vehicle.

This section describes our findings regarding changes in inspiratory drive, defined as the central urge to inhale calculated by the ratio of tidal volume and inspiratory duration (TV/Ti), and the changes in expiratory drive defined as the central urge to exhale as calculated by the ratio of tidal volume and expiratory duration (TV/Te). As seen in Figure 7, the injection of atropine elicited substantial and sustained increases in InspD (Panel A) and ExpD (Panel B). The morphine-induced decreases in InspD and ExpD seen in vehicle-treated rats were markedly reduced in the atropine-treated rats. Each dose of L-CYSee elicited substantial increases in InspD and ExpD in saline-treated rats. The responses elicited by the second injection were markedly diminished in atropine-treated rats. The HH-induced increases in InspD and ExpD occurred in parallel in the two groups, and the lower plateau levels reached upon return to room air were similar in both groups. As summarized in Panels D and E, atropine elicited substantial increases in InspD and ExpD with the major observation being that the L-CYSee-induced changes in InspD and ExpD were markedly diminished in the atropine-treated rats.

#### 2.2.8. Non-Eupneic Breathing Index (NEBI) and NEBI Corrected for Changes in Freq (NEBI/Freq)

**Figure 8:**
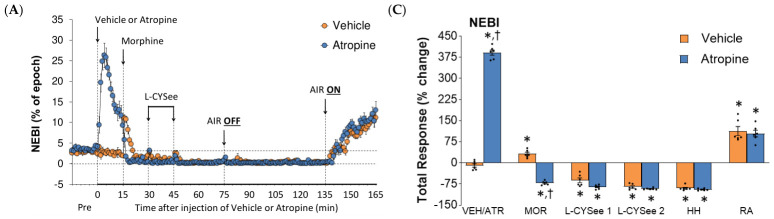

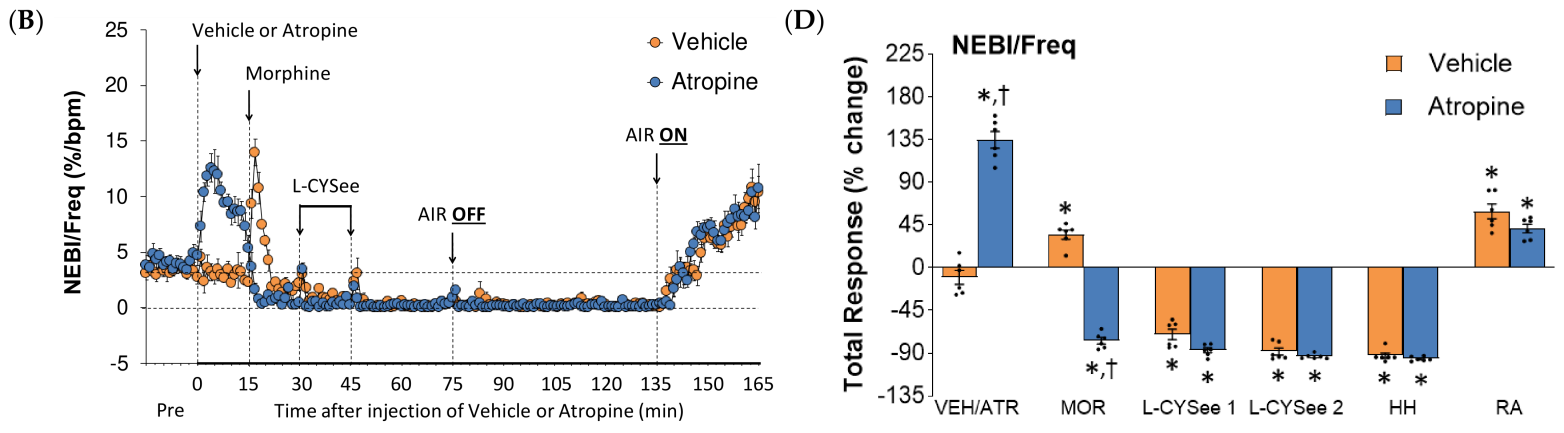
Values recorded for non-eupneic breathing index (NEBI) (Panel (**A**)) and NEBI corrected for frequency of breathing (NEBI/Freq) (Panel (**B**)) before (Pre), after injection of vehicle (VEH) or atropine (1 mg/kg, IV), then morphine (10 mg/kg, IV), and finally subsequent injections of L-cysteine ethyl ester (L-CYSee, 2 × 500 μmol/kg, IV) followed by a hypoxic-hypercapnic gas challenge (AIR OFF) and return to room-air (AIR ON). A summary of the total changes in NEBI and NEBI/Freq during each phase of the study is presented in Panels (**C**,**D**), respectively (ATR—atropine; HH—hypoxic-hypercapnic gas challenge; RA—return to room-air). The data are presented as mean ± SEM. There were six rats in each group. * *p* < 0.05, significant change from Pre-values. ^†^ *p* < 0.05, atropine versus vehicle.

This section describes our findings regarding changes in the non-eupneic breathing index (NEBI), defined as the percentage of non-eupneic breaths in each 1 min recording period, and NEBI corrected for breathing frequency in each epoch (NEBI/Freq) to determine whether breathing rate affects NEBI. As seen in Figure 8, atropine elicited substantial and sustained increases in NEBI (Panel A) and NEBI/Freq (Panel B). Morphine induced substantial decreases in NEBI and NEBI/Freq, and neither the L-CYSee injections or HH-challenge affected these changes. The return to room air elicited marked increases in NEBI and NEBI/Freq that were similar in both groups. As summarized in Panels D and E, atropine elicited marked increases in NEBI and NEBI/Freq, with major observations being that the morphine-induced changes in these parameters were exacerbated by atropine.

### 2.3. Righting Reflex Studies

The sedation times (return of the righting reflex in the four treatment groups are presented in Table 1. The morphine induced loss of righting reflex in vehicle-treated rats recovered in 79.1 ± 4.4 min, mean ± SEM). This recovery time was shortened to 39.6 ± 2.9 min by atropine. L-CYSee did not affect the morphine-induced loss of righting reflex (69.4 ± 11.6 min). However, the recovery of the righting reflex was shortest (16.8 ± 1.5 min) in the morphine group of rats that received atropine and L-CYSee.

## 3. Discussion

### 3.1. Ventilatory Responses Elicited by Atropine

The injection of atropine (1.0 mg/kg, IV) elicited an array of changes in ventilatory parameters in naïve adult male Sprague-Dawley rats. A summary of the changes elicited by atropine in naïve rats (present studies) and in those that received a prior injection of morphine [29] is given in Supplemental Table S4. In naïve rats, atropine elicited a substantial and sustained increase in Freq and a lesser, but sustained, decrease in TV, such that MV rose appreciably. This novel finding may be due to cholinergic receptor mechanisms having opposite effects in neural pathways regulating Freq and TV, although we have not found published evidence to support this possibility. The atropine-induced tachypnea may be driven by block of muscarinic receptors in the pre-Bötzinger complex in the medulla oblongata (primary generator of inspiratory breathing rhythm), while the atropine-induced decrease of TV may be due to a combination of reduced pre-motor activity in the pons and/or reduced respiratory motor output of spinal motor neurons [45]. With respect to atropine-induced increases in Freq, Muere et al. [46] reported that microdialysis of atropine near the pre-Botzinger complex increases breathing frequency more during wakefulness than during NREM sleep in goats.

Other evidence for a role of central muscarinic receptors in ventilatory control processes includes that (a) cholinergic muscarinic receptor neurotransmission activates parafacial respiratory neurons that recruit abdominal muscles to enhance active expiratory flow [47], (b) pontine cholinergic mechanisms play roles in ventilatory control processes [48,49], (c) transition from inspiration to expiration depends on active interplay between intrinsic non-cholinergic neural networks within the pons and input from slowly adapting pulmonary stretch receptors [50], and (d) these ventilatory control systems are influenced by the intermediate reticular nucleus, which co-ordinates coupling of respiratory-sympathetic nerve activity, swallowing and post-inspiratory activity [51]. It is possible that (a) the atropine-mediated tachypnea is caused by a neural reflex designed to limit the influence of the tachypnea on MV, and (b) the atropine-mediated reduction in TV elicits a neural reflex to increase Freq. The possibility of atropine-mediated fall in TV is a reflex adjustment to the tachypnea, as supported by our findings that atropine increased PIF, PEF, and EF_50_, accompanied by decreases in ventilatory mechanical timing events, namely, Rpef, relaxation time, expiratory delay (Te-RT) and apneic Pause [(Te/RT)-1]. The finding that atropine elicited sustained increases in inspiratory drive and expiratory drive suggests that the overall role of muscarinic receptor-mediated events is the suppression of ventilatory drive while the substantial increase in NEBI and NEBI/Freq produced by atropine suggests that cholinergic–muscarinic receptor pathways in the brain [52,53] and carotid body [39,54] play a vital role in suppressing non-eupneic breathing events that would include disordered breaths, apneas and type 1 and 2 sighs [see 4].

### 3.2. Ventilatory Responses Elicited by Morphine

The bolus injection of morphine (10 mg/kg, IV) produced an array of ventilatory responses in adult male vehicle-treated Sprague-Dawley rats that were consistent with those we reported previously [4,13,14,29]. In brief, morphine elicited a depression of breathing, including sustained decreases in TV, MV, PIF, PEF and inspiratory drive associated with marked increases in EIP, EEP, EF_50_, and Rpef. Most effects of morphine were not directly altered by atropine (changes in baselines by atropine complicate this assertion), although final baselines after injection of morphine reached similar levels in vehicle- or atropine-treated rats. In contrast, the morphine-induced increases in EIP, EEP and Rpef were clearly diminished in atropine-treated rats. As such, it appears that muscarinic receptors are exquisitely involved in regulating the process that controls end-inspiratory and end-expiratory pauses as well as the dynamic increases in PEF. A full description of the effects of atropine on the ventilatory responses elicited by morphine and the meaning of these effects is described in detail in our companion manuscript [29].

### 3.3. Ventilatory Responses Elicited by L-CYSee

As expected, this study found that subsequent injections of L-CYSee (2 × 500 μmol/kg, IV given 15 min apart) substantially reversed the effects of morphine on ventilatory parameters in vehicle-treated rats [14]. These findings are supported by evidence that L-CYSee, L-cysteine methyl ester, D-cysteine ethyl ester, D-cysteine di(m)ethyl ester, L-glutathione ethyl ester and the S-nitrosothiol, S-nitroso-L-cysteine, overcome opioid (morphine or fentanyl)-induced respiratory depression, alveolar gas exchange and arterial blood-gas chemistry in rats without reducing opioid analgesia see [4,14]. Regarding potential mechanisms by which L-CYSee and related analogues overcome OIRD, the present study shows here that the ability of L-CYSee to overcome the adverse effects of morphine, for example, Freq, TV and MV, were markedly diminished in atropine-treated rats. As such, a major mechanism by which L-CYSee overcomes morphine-induced ventilatory suppression may be via direct and/or indirect activation of muscarinic receptor-dependent signaling process in the central nervous system and other relevant structures such as the carotid bodies and musculoskeletal system. Both brainstem and forebrain structures may be involved since activation of muscarinic receptor-dependent mechanisms in medulla oblongata coordinates inspiratory and expiratory timing events in goats [55], muscarinic receptor stimulation in the brainstem reverses fentanyl-induced respiratory depression in anesthetized rats [56], and forebrain muscarinic receptors play a vital role in the ability of morphine to regulate breathing in fetal lambs [57]. We have demonstrated that low doses of morphine actually stimulate breathing in rats and that fentanyl activates opposing opioid (inhibitory) and non-opioid receptor (excitatory) systems that control breathing in rats [4,14]. It would seem possible that the excitatory non-opioid receptor system is a cholinergic–muscarinic receptor system in central and peripheral neural circuitry. Activation of muscarinic receptors may therefore play a vital role in the ability of thiol analogues such as L-CYSee to reverse OIRD.

### 3.4. Ventilatory Responses to HH-Challenge

As expected, the HH-challenge elicited changes in ventilatory parameters in morphine-treated rats that were reduced compared to naïve rats [29,41]. Getsy et al. [29] reported that several ventilatory responses during HH-challenge in morphine-treated rats were blunted by atropine suggesting roles for muscarinic receptors in specific aspects of the mechanisms responsive to HH-challenge. The ability of L-CYSee to enhance ventilatory responses during HH-challenge in morphine-treated rats was reduced by atropine suggesting that L-CYSee may activates muscarinic-receptor process in brain [48,49,50,58,59] and carotid body [20,21,22,23] structures driving the ventilatory responses to HH-challenge. Our findings are consistent with evidence that endogenous opiates are involved in regulating breathing by interactions with CO_2_-sensitive cholinergic neurons on the caudal ventral medullary surface [60]. Moreover, our findings are also in agreement with evidence that cholinergic muscarinic receptor mechanisms within the medial prefrontal cortex influence state-dependent ventilatory response to hypercapnia [61].

### 3.5. Ventilatory Responses on Return to Room Air

The neurophysiological/neurochemical mechanisms responsible for the ventilatory responses that occur upon return to room air after hypoxic, hypercapnic and HH-challenges have received attention [4,29,41]. The carotid body-carotid sinus nerve complex is vital to the expression of the responses and that redox status, nitric oxide synthase and S-nitrosothiols also play key roles [4,29,41]. The ability of morphine to inhibit or exaggerate many of these return to room air responses has been described [4,29,41], as has the ability of atropine to modulate HH-challenge responses in vehicle- or morphine-treated rats [29]. In the present study, there were few interactions between atropine and L-CYSee in modulating return to room-air responses in morphine-treated rats. However, atropine did augment the rise in Rpef, whereas it decreased the rise in EIP. The rise in Rpef was similar in size and duration to rats that received morphine + atropine only [29] and so cannot be attributed to an interaction between L-CYSee and muscarinic receptors, although cholinergic–muscarinic processes would normally suppress the rate of reaching PEF. With respect to the decrease in EIP, since the transition from inspiration to expiration relies on interplay between intrinsic non-cholinergic neural networks in the pons [50], it appears that cholinergic–muscarinic processes outside the pons reduce the pause between inspiration and the start of expiration. In contrast, atropine caused a rise in EEP that was likely due to augmenting the actions of L-CYSee since this effect did not occur in rats treated with morphine + atropine only [29]. As such, the ability of L-CYSee to suppress the pause between the end of inspiration and the beginning of expiration seems to rely on muscarinic receptor processes in neural signalling pathways.

### 3.6. Role of Muscarinic Receptors in the Effects of L-CYSee on Morphine-Induced Sedation

As reported in our companion manuscript [29], atropine markedly reduced the time of recovery of the righting reflex in morphine-treated rats. The interpretation of these findings is somewhat complicated since if the sedative effects of morphine were due to the release of neural acetylcholine and activation of muscarinic receptors, then atropine would be expected to arouse the rats more or less immediately rather than shortening the duration of sedation from 79.1 ± 4.4 min to 39.6 ± 2.9 min. It is evident that muscarinic receptor stimulation does not initiate the morphine-sedation but becomes increasingly more important to the maintenance of the sedation. The ability of atropine to affect the sedative effects of morphine is consistent with findings that the muscarinic receptor antagonist affects some behavioral manifestations of morphine [62,63,64,65,66,67]. That L-CYSee did not modify the sedative effects of morphine does not mean a lack of activity since L-CYSee greatly shortened the sedative effects of morphine in the atropine-treated rats to values below those that received atropine and morphine only. It could be tentatively stated that L-CYSee has the ability to diminish the sedative actions of morphine, but concomitant activation of cholinergic pathways prevents this from occurring. More specifically, atropine uncovered the ability of L-CYSee to reduce the sedative effects of morphine by mechanisms yet to be determined.

### 3.7. Conclusions and Limitations

Many L-CYSee-induced effects on the ventilatory actions of morphine in male rats seemed to be dependent upon activation of cholinergic muscarinic receptors. Potential mechanisms include that (a) L-CYSee directly activates muscarinic receptors and/or muscarinic receptor signaling events by processes that are augmented by concomitant opioid receptor-mediated signaling events, and (b) L-CYSee activates cholinergic neurotransmission/activation of muscarinic receptors within ventilatory control structures. Questions arise as to whether (a) L-CYSee activates muscarinic-receptor signaling in female rats because of potential sex differences in neurochemical/neurobiological processes that control breathing [68,69] although sex differences in muscarinic-receptor signaling in ventilatory control pathways have not been reported, and (b) whether the ability of L-CYSee analogues such as L-CYSme, D-CYSee, to overcome morphine OIRD also involve muscarinic receptors in male and female rats. The studies need to be replicated with the peripherally restricted muscarinic receptor antagonist, methylatropine [30], to determine the specific roles of peripheral (e.g., carotid bodies) muscarinic receptors in the expression of ventilatory actions of L-CYSee. Studies to determine which of the five muscarinic receptors (M1-M5) sensitive to atropine [30] should be done to clarify the role of specific muscarinic receptor subtypes in the ability of L-CYSee to overcome the ventilatory depressant effects of morphine. The possibility that L-CYSee activates muscarinic receptors to overcome OIRD requires mechanistic support rather than simple reliance on atropine studies before a firm conclusion can be reached. Evidence suggesting that atropine affects the activities of cholinergic nicotinic receptors [70], alpha-adrenoceptors [71], and phosphodiesterase type 4 [72] will be taken into consideration when attempting to clarify the mechanisms of action of L-CYSee. Finally, to our knowledge, there is no evidence to support the possibility that L-CYSee is an endogenous compound. Moreover, L-CYSee does not appear to be available for human use at present.

## 4. Materials and Methods

### 4.1. Permissions, Rats, Surgeries

The studies were done in accordance with the NIH Guide for Care and Use of Laboratory Animals (NIH Publication No. 80-23) revised in 1996, and in compliance with ARRIVE (Animal Research: Reporting of In Vivo Experiments) guidelines (https://arriveguidelines.org/, accessed on 12 January 2023). All studies were approved by the Animal Care and Use Committees of the University of Virginia, Galleon Pharmaceuticals, Inc., and Case Western Reserve University. Adult male Sprague Dawley rats from Harlan Industries (Madison, WI, USA) were given 6 days of recovery from transport and implanted with jugular vein catheters under 2–3% isoflurane [4,13,14]. Catheters were flushed with 0.3 mL of phosphate-buffered saline (0.1 M, pH 7.4) 2–3 h before each study. Morphine sulfate was from Baxter Healthcare Corporation (Deerfield, IL, USA) and given to us by our Animal Resources Center. Atropine sulfate hydrate (A0257-5G) and L-CYSee (L-Cysteine ethyl ester hydrochloride, C121908-25G) were from Sigma-Aldrich (St. Louis, MO, USA). The doses of morphine sulfate, atropine sulfate hydrate and L-CYSee are expressed in terms of base compound, namely, morphine (10 mg/kg, IV), atropine (1 mg/kg, IV) and L-CYSee (500 μmol/kg, IV; 141.71 mg/kg, IV). The studies were done in a quiet room with a relative humidity of 49 ± 2% and a temperature of 21.3 ± 0.2 °C. Plethysmography sessions were done by an investigator who gave the vehicle and test drugs. The syringes with vehicle or test drug were made up by one investigator, and another performing the experiment was blinded to the protocol. Data files from each experiment were collated and analyzed by another investigator. These studies were performed in two separate cohorts of vehicle-treated (three and three rats) or atropine-treated rats (three and three rats) done 3 months apart.

### 4.2. Protocols for Measurement of Ventilatory Parameters

Ventilatory parameters were continuously recorded in unrestrained, unanesthetized rats via whole-body plethysmography (PLY3223; Data Sciences International, St. Paul, MN, USA) [4,13,14]. The directly recorded and derived parameters are defined in Table S1. These parameters are: Freq—frequency of breathing; TV—tidal volume; MV—minute ventilation; Ti—inspiratory time; Te—expiratory time; Te/Ti (respiratory quotient); EIP—end inspiratory pause; EEP—end expiratory pause; PIF—peak inspiratory flow; PEF—peak expiratory flow; PEF/PIF (flow balance); Rpef—rate of achieving PEF; EF_50_—expiratory flow at 50% expired TV; RT—relaxation time; expiratory delay—Te-RT; AP—apneic pause, (Te/RT)-1; TV/TI—inspiratory drive; TV/Te—expiratory drive; NEBI, non-eupneic breathing index and NEBI/Freq—NEBI corrected for Freq. A diagram of the relationships between directly recorded parameters and especially the relationship between Te and RT is provided in Figure S1 and explained by Lomask [73]. On the day of the study, each rat was placed in an individual plethysmography chamber and allowed at least 60 min to acclimatize, during which resting (baseline, pre) ventilatory parameters were accurately defined.

One group of rats received an injection of vehicle (saline, 300 μL/kg) and after 15 min they received an injection of morphine (10 mg/kg, IV). After another 15 min the rats received two injections of L-CYSee (500 μmol/kg, IV) given 15 min apart. After 30 min, the rats were subjected to a rebreathing HH-challenge [51,55,56] for 60 min followed by return to room air for 30 min. A second group was injected with atropine (1.0 mg/kg, IV) followed by identical treatments as above. The body weights of the two groups were similar to one another (Table S2), and so volume parameters (e.g., TV, PIF, PEF, and EF_50_) are shown without body weight corrections. FinePointe (DSI) software(v2.9.0) constantly corrected digitized ventilatory values originating from the raw waveforms for alterations in chamber humidity and chamber temperature. Pressure changes associated with the respiratory waveforms were converted to volumes using the installed proprietary software algorithms. Factoring chamber humidity and temperature, cycle analyzers (DSI, St. Paul, MN, USA) filtered the acquired signals, and FinePointe algorithms generated an array of box flow data that identified a waveform segment as an acceptable breath. From that data, maximum and minimum values were determined. Flows at this juncture were box-flow signals, and from these, maximum and minimum and box flow values were determined and multiplied by a compensation factor provided by the algorithm to produce TV, PIF, PEF, and EF_50_ values used to determine non-eupneic breathing events expressed as non-eupneic breathing index (NEBI), expressed as percent of non-eupneic breathing events per epoch [4,13,14].

### 4.3. Righting Reflex as an Index of Morphine-Induced Sedation

Four groups of adult male Sprague Dawley rats (see Supplemental Table S3 for ages and body weights) were used to evaluate the effects of atropine and L-CYSee on the duration of the loss of the righting reflex (i.e., inability to stand on all 4 legs) elicited by the injection of morphine [4,13,14]. The rats were placed into individual open plastic chambers to determine the length of time the rats took to recover from the morphine-induced loss of the righting reflex. The external end of the jugular vein catheter was connected to tubing connected to a syringe to injection drugs. The rats were given 90 min to acclimatize to the chambers. The time when the rat stood on all four paws for at least 12 s (the time we established as the time at which 100% of the rats retain an upright stance) after injection of morphine was taken as the time of recovery of the righting reflex [4,13,14]. The first group of rats received an injection of vehicle (saline, 100 μL/kg, IV) and after 5 min an injection of morphine (10 mg/kg, IV) and after 5 min they received an injection of vehicle (saline). The second group received an injection of atropine (1.0 mg/kg, IV) and after 5 min an injection of morphine (10 mg/kg, IV) and after 5 min an injection of vehicle (saline). The third group received vehicle (saline, 100 μL/kg, IV), and after 5 min, an injection of morphine (10 mg/kg, IV) and after 5 min, they received L-CYSee (500 μg/kg, IV). The fourth group received an injection of atropine (1.0 mg/kg, IV) and after 5 min an injection of morphine (10 mg/kg, IV), and after 5 min they were injected with L-CYSee (500 μmol/kg, IV). Recovery of the righting reflex was determined by two investigators blinded to the protocols.

### 4.4. Data Analyses

All data are presented as mean ± SEM (and SD in some cases) and were analyzed by one-way and two-way ANOVA, with Bonferroni corrections for multiple comparisons between means, using the error mean square terms generated by the ANOVA analyses [4,13,14]. A *p* < 0.05 value was the initial level of significance that was modified according to the number of between-mean comparisons [4,13,14]. The modified *t*-statistic for two groups, for instance, is t = (mean group 1 − mean group 2)/[s × (1/n_1_ + 1/n_2_)^1/2^], where s^2^ = mean square within groups term from the ANOVA and n_1_ and n_2_ are the numbers in each group being compared. Statistics were done with GraphPad Prism (version 9.5.1) software (GraphPad Software, Inc., La Jolla, CA, USA).

## Figures and Tables

**Table 1:** Sedation times in the morphine-injected groups.

Treatment Group	Actual Values (min)	Mean ± SD ± SEM
Vehicle + morphine + vehicle	63, 65, 65, 78, 81, 84, 88, 96, 92	79.1 ± 12.3 ± 4.4
Atropine + morphine + vehicle	30, 31, 31, 37, 42, 43, 43, 44, 55	39.6 ± 8.2 ± 2.9 *
Vehicle + morphine + L-CYSee	56, 57, 59, 61, 69, 75, 81, 82, 85	69.4 ± 4.1 ± 11.6
Atropine + morphine + L-CYSee	17, 11, 15, 22, 18, 12, 13, 21, 22	16.8 ± 4.3 ± 1.5 *^,†^
**ANOVA**		***p* value**
F_3,32_ = 78.4		<0.0001
Vehicle + morphine + vehicle versus Atropine + morphine + vehicle		<0.0001
Vehicle + morphine + vehicle versus Vehicle + morphine + L-CYSee		=0.17
Vehicle + morphine + vehicle versus Atropine + morphine + L-CYSee		=0.0001
Atropine + morphine + vehicle versus Vehicle + morphine + L-CYSee		<0.00001
Atropine + morphine + vehicle versus Atropine + morphine + L-CYSee		=0.0001
Vehicle + morphine + L-CYSee versus Atropine + morphine + L-CYSee		<0.0001

* significant difference *from Vehicle + morphine + vehicle*. ^†^ Atropine + morphine + L-CYSee versus Atropine + morphine + vehicle.

## Data Availability

The original contributions presented in this study are included in the article/Supplementary Materials. Further inquiries can be directed to the corresponding author.

## Supplementary Materials

The following supporting information can be downloaded at: https://www.mdpi.com/article/10.3390/ph19071125/s1, Figure S1: Relationships between peak inspiratory flow (PIF), peak expiratory flow (PEF), relaxation time (RT) and expiratory time (Te); Table S1: Definition of ventilatory parameters described in this study; Table S2: Baseline values in the two groups of rats used for the ventilatory studies; Table S3: Ages and body weights of the four groups of rats used for the righting-reflex studies; Table S4: Comparison of the effects of atropine in naïve and morphine-treated rats.

## Abbreviations

OIRD—opioid-induced respiratory depression; L-CYSee—L-cysteine ethyl ester. HH-challenge—hypoxic-hypercapnic challenge; Freq—frequency of breathing; TV—tidal volume; MV—minute ventilation; Ti—inspiratory time; Te—expiratory time; Te/Ti (respiratory quotient); EIP—end inspiratory pause; EEP—end expiratory pause; PIF—peak inspiratory flow; PEF—peak expiratory flow; PEF/PIF (flow balance) Rpef, rate of achieving PEF; EF_50_—expiratory flow at 50% expired TV; RT—relaxation time; expiratory delay—Te-RT; AP—apneic pause, (Te/RT)-1; TV/TI—inspiratory drive; TV/Te—expiratory drive; NEBI—non-eupneic breathing index, and NEBI/Freq—NEBI corrected for the frequency of breathing.

## References

[1] Algera M.H., Kamp J., van der Schrier R., van Velzen M., Niesters M., Aarts L., Dahan A., Olofsen E. (2019). Opioid-induced respiratory depression in humans: A review of pharmacokinetic-pharmacodynamic modelling of reversal. Br. J. Anaesth..

[2] Imam M.Z., Kuo A., Smith M.T. (2020). Countering opioid-induced respiratory depression by non-opioids that are respiratory stimulants. F1000Research.

[3] Dahan A., Aarts L., Smith T.W. (2010). Incidence, Reversal, and Prevention of Opioid-induced Respiratory Depression. Anesthesiology.

[4] Getsy P.M., Baby S.M., May W.J., Bates J.N., Ellis C.R., Feasel M.G., Wilson C.G., Lewis T.H.J., Gaston B., Hsieh Y.H. (2022). L-cysteine methyl ester overcomes the deleterious effects of morphine on ventilatory parameters and arterial blood-gas chemistry in unanesthetized rats. Front. Pharmacol..

[5] Trivedi M., Shah J., Hodgson N., Byun H.M., Deth R. (2014). Morphine induces redox-based changes in global DNA methylation and retrotransposon transcription by inhibition of excitatory amino acid transporter type 3-mediated cysteine uptake. Mol. Pharmacol..

[6] Trivedi M.S., Deth R. (2015). Redox-based epigenetic status in drug addiction: A potential contributor to gene priming and a mechanistic rationale for metabolic intervention. Front. Neurosci..

[7] Chung H.S., Wang S.B., Venkatraman V., Murray C.I., Van Eyk J.E. (2013). Cysteine oxidative posttranslational modifications: Emerging regulation in the cardiovascular system. Circ. Res..

[8] Pace N.J., Weerapana E. (2013). Diverse functional roles of reactive cysteines. ACS Chem. Biol..

[9] Santos A.I., Martínez-Ruiz A., Araújo I.M. (2015). S-nitrosation and neuronal plasticity. Br. J. Pharmacol..

[10] Paul B.D., Sbodio J.I., Snyder S.H. (2018). Cysteine Metabolism in Neuronal Redox Homeostasis. Trends Pharmacol. Sci..

[11] Bak D.W., Bechtel T.J., Falco J.A., Weerapana E. (2019). Cysteine reactivity across the subcellular universe. Curr. Opin. Chem. Biol..

[12] Garrido Ruiz D., Sandoval-Perez A., Rangarajan A.V., Gunderson E.L., Jacobson M.P. (2022). Cysteine Oxidation in Proteins: Structure, Biophysics, and Simulation. Biochemistry.

[13] Lewis T.H.J., May W.J., Young A.P., Bates J.N., Baby S.M., Getsy P.M., Ryan R.M., Hsieh Y.H., Seckler J.M., Lewis S.J. (2022). The ventilatory depressant actions but not the antinociceptive effects of morphine are blunted in rats receiving intravenous infusion of L-cysteine ethyl ester. Biomed. Pharmacother..

[14] Baby S.M., May W.J., Young A.P., Wilson C.G., Getsy P.M., Coffee G.A., Lewis T.H.J., Hsieh Y.H., Bates J.N., Lewis S.J. (2024). L-cysteine ethylester reverses the adverse effects of morphine on breathing and arterial blood-gas chemistry while minimally affecting antinociception in unanesthetized rats. Biomed. Pharmacother..

[15] Kubin L., Fenik V. (2004). Pontine cholinergic mechanisms and their impact on respiratory regulation. Respir. Physiol. Neurobiol..

[16] Bonis J.M., Neumueller S.E., Krause K.L., Kiner T., Smith A., Marshall B.D., Qian B., Pan L.G., Forster H.V. (2010). A role for the Kolliker-Fuse nucleus in cholinergic modulation of breathing at night during wakefulness and NREM sleep. J. Appl. Physiol. (1985).

[17] Boutin R.C., Alsahafi Z., Pagliardini S. (2017). Cholinergic modulation of the parafacial respiratory group. J. Physiol..

[18] Moreira T.S., Sobrinho C.R., Falquetto B., Oliveira L.M., Lima J.D., Mulkey D.K., Takakura A.C. (2021). The retrotrapezoid nucleus and the neuromodulation of breathing. J. Neurophysiol..

[19] Biancardi V., Yang X., Ding X., Passi D., Funk G.D., Pagliardini S. (2023). Cholinergic projections to the preBötzinger complex. J. Comp. Neurol..

[20] Eyzaguirre C., Zapata P. (1968). The release of acetylcholine from carotid body tissues. Further study on the effects of acetylcholine and cholinergic blocking agents on the chemosensory discharge. J. Physiol..

[21] Fidone S., Sato A., Eyzaguirre C. (1968). Acetylcholine activation of carotid body chemoreceptor A fibers. Brain Res..

[22] Nurse C.A., Zhang M. (1999). Acetylcholine contributes to hypoxic chemotransmission in co-cultures of rat type 1 cells and petrosal neurons. Respir. Physiol..

[23] Kim D.K., Prabhakar N.R., Kumar G.K. (2004). Acetylcholine release from the carotid body by hypoxia: Evidence for the involvement of autoinhibitory receptors. J. Appl. Physiol. (1985).

[24] Elmalem E., Chorev M., Weinstock M. (1991). Antagonism of morphine-induced respiratory depression by novel anticholinesterase agents. Neuropharmacology.

[25] Tsujita M., Sakuraba S., Kuribayashi J., Hosokawa Y., Hatori E., Okada Y., Kashiwagi M., Takeda J., Kuwana S. (2007). Antagonism of morphine-induced central respiratory depression by donepezil in the anesthetized rabbit. Biol. Res..

[26] Sakuraba S., Tsujita M., Arisaka H., Takeda J., Yoshida K., Kuwana S. (2009). Donepezil reverses buprenorphine-induced central respiratory depression in anesthetized rabbits. Biol. Res..

[27] Ren J., Ding X., Greer J.J. (2019). Activating α4β2 Nicotinic Acetylcholine Receptors Alleviates Fentanyl-induced Respiratory Depression in Rats. Anesthesiology.

[28] Ren J., Ding X., Greer J.J. (2020). Countering Opioid-induced Respiratory Depression in Male Rats with Nicotinic Acetylcholine Receptor Partial Agonists Varenicline and ABT 594. Anesthesiology.

[29] Getsy P.M., May W.J., Baby S.M., Coffee G.A., Qiu Y., Cheng F., Bates J.N., Lewis S.J. (2026). Role of muscarinic receptor signaling processes in specific aspects of morphine-induced respiratory depression in rats. Pharmaceuticals.

[30] Svoboda J., Popelikova A., Stuchlik A. (2017). Drugs Interfering with Muscarinic Acetylcholine Receptors and Their Effects on Place Navigation. Front. Psychiatry..

[31] Picciotto M.R., Higley M.J., Mineur Y.S. (2012). Acetylcholine as a neuromodulator: Cholinergic signaling shapes nervous system function and behavior. Neuron.

[32] Jiang S., Li Y., Zhang C., Zhao Y., Bu G., Xu H., Zhang Y.W. (2014). M1 muscarinic acetylcholine receptor in Alzheimer’s disease. Neurosci. Bull..

[33] Lakstygal A.M., Kolesnikova T.O., Khatsko S.L., Zabegalov K.N., Volgin A.D., Demin K.A., Shevyrin V.A., Wappler-Guzzetta E.A., Kalueff A.V. (2019). DARK Classics in Chemical Neuroscience: Atropine, Scopolamine, and Other Anticholinergic Deliriant Hallucinogens. ACS Chem. Neurosci..

[34] Zhang W., Basile A.S., Gomeza J., Volpicelli L.A., Levey A.I., Wess J. (2022). Characterization of central inhibitory muscarinic autoreceptors by the use of muscarinic acetylcholine receptor knock-out mice. J. Neurosci..

[35] Dinger B.G., Hirano T., Fidone S.J. (1986). Autoradiographic localization of muscarinic receptors in rabbit carotid body. Brain Res..

[36] Dasso L.L., Buckler K.J., Vaughan-Jones R.D. (1997). Muscarinic and nicotinic receptors raise intracellular Ca^2+^ levels in rat carotid body type I cells. J. Physiol..

[37] Shirahata M., Hirasawa S., Okumura M., Mendoza J.A., Okumura A., Balbir A., Fitzgerald R.S. (2004). Identification of M1 and M2 muscarinic acetylcholine receptors in the cat carotid body chemosensory system. Neuroscience.

[38] Bairam A., Joseph V., Lajeunesse Y., Kinkead R. (2006). Developmental pattern of M1 and M2 muscarinic gene expression and receptor levels in cat carotid body, petrosal and superior cervical ganglion. Neuroscience.

[39] Ortiz F.C., Varas R. (2010). Muscarinic modulation of TASK-like background potassium channel in rat carotid body chemoreceptor cells. Brain Res..

[40] Haga T. (2013). Molecular properties of muscarinic acetylcholine receptors. Proc. Jpn. Acad. Ser. B Phys. Biol. Sci..

[41] Getsy P.M., Coffee G.A., May W.J., Baby S.M., Bates J.N., Lewis S.J. (2024). The Reducing Agent Dithiothreitol Modulates the Ventilatory Responses That Occur in Freely Moving Rats during and following a Hypoxic-Hypercapnic Challenge. Antioxidants.

[42] Matthew C.B., Thomas G.J., Hubbard R.W., Francesconi R.P. (1988). Intramuscular and intravenous atropine: Comparison of effects in the heat-stressed rat. Aviat. Space Environ. Med..

[43] Pagniez F., Valentin J.P., Vieu S., Colpaert F.C., John G.W. (1998). Pharmacological analysis of the haemodynamic effects of 5-HT1B/D receptor agonists in the normotensive rat. Br. J. Pharmacol..

[44] Nakajima Y., Tsujimura T., Tsutsui Y., Chotirungsan T., Kawada S., Dewa N., Magara J., Inoue M. (2023). Atropine facilitates water-evoked swallows via central muscarinic receptors in anesthetized rats. Am. J. Physiol. Gastrointest. Liver Physiol..

[45] Doi A., Ramirez J.M. (2008). Neuromodulation and the orchestration of the respiratory rhythm. Respir. Physiol. Neurobiol..

[46] Muere C., Neumueller S., Miller J., Olesiak S., Hodges M.R., Pan L., Forster H.V. (2013). Atropine microdialysis within or near the pre-Botzinger Complex increases breathing frequency more during wakefulness than during NREM sleep. J. Appl. Physiol. (1985).

[47] Silva J.N., Oliveira L.M., Souza F.C., Moreira T.S., Takakura A.C. (2019). Distinct pathways to the parafacial respiratory group to trigger active expiration in adult rats. Am. J. Physiol. Lung Cell Mol. Physiol..

[48] Sergeeva L.I., Kuz’mina V.E. (1994). Participation of cholinergic systems in the bulbar mechanisms of the regulation of breathing. Neurosci. Behav. Physiol..

[49] Rukhadze I., Kubin L. (2007). Mesopontine cholinergic projections to the hypoglossal motor nucleus. Neurosci. Lett..

[50] Bonham A.C. (1995). Neurotransmitters in the CNS control of breathing. Respir. Physiol..

[51] Toor R.U.A.S., Sun Q.J., Kumar N.N., Le S., Hildreth C.M., Phillips J.K., McMullan S. (2019). Neurons in the Intermediate Reticular Nucleus Coordinate Postinspiratory Activity, Swallowing, and Respiratory-Sympathetic Coupling in the Rat. J. Neurosci..

[52] Boccalini C., Perani D., Garibotto V. (2025). Memory network and cognitive reserve are associated with preserved and stimulated cholinergic neurotransmission. Handb. Clin. Neurol..

[53] Fisher A., Levey A.I. (2025). CNS muscarinic receptors and muscarinic receptor agonists in Alzheimer disease treatment. Handb. Clin. Neurol..

[54] Lazarov N.E., Atanasova D.Y. (2023). Neurochemical Anatomy of the Mammalian Carotid Body. Adv. Anat. Embryol. Cell Biol..

[55] Forster H.V., Julius H. (2018). Comroe Distinguished Lecture: Interdependence of neuromodulators in the control of breathing. J. Appl. Physiol. (1985).

[56] Willette R.N., Doorley B.M., Sapru H.N. (1987). Activation of cholinergic mechanisms in the medulla oblongata reverse intravenous opioid-induced respiratory depression. J. Pharmacol. Exp. Ther..

[57] Szeto H.H., Cheng P.Y., Dwyer G., Decena J.A., Wu D.L., Cheng Y. (1991). Morphine-induced stimulation of fetal breathing: Role of mu 1-receptors and central muscarinic pathways. Am. J. Physiol..

[58] Pedersen O.F. (1997). The Peak Flow Working Group: Physiological determinants of peak expiratory flow. Eur. Respir. J..

[59] Wu M., Haxhiu M.A., Johnson S.M. (2005). Hypercapnic and hypoxic responses require intact neural transmission from the pre-Bötzinger complex. Respir. Physiol. Neurobiol..

[60] Trouth C.O., Millis R.M., Bernard D.G., Pan Y., Whittaker J.A., Archer P.W. (1993). Cholinergic-opioid interactions at brainstem respiratory chemosensitive areas in cats. Neurotoxicology.

[61] Lydic R., Baghdoyan H.A., Wertz R., White D.P. (1991). Cholinergic reticular mechanisms influence state-dependent ventilatory response to hypercapnia. Am. J. Physiol..

[62] Horita A., Carino M.A., Chinn C. (1988). Codeine produces a cholinergically mediated analeptic effect in rats and rabbits. Pharmacol. Biochem. Behav..

[63] Horita A., Carino M.A., Chinn C. (1999). Fentanyl produces cholinergically-mediated analeptic and EEG arousal effects in rats. Neuropharmacology.

[64] Zarrindast M.R., Samadi P., Haeri-Rohani A., Moazami N., Shafizadeh M. (2002). Nicotine potentiation of morphine-induced catalepsy in mice. Pharmacol. Biochem. Behav..

[65] Zarrindast M.R., Fattahi Z., Rostami P., Rezayof A. (2005). Role of the cholinergic system in the rat basolateral amygdala on morphine-induced conditioned place preference. Pharmacol. Biochem. Behav..

[66] Rezayof A., Zatali H., Haeri-Rohani A., Zarrindast M.R. (2006). Dorsal hippocampal muscarinic and nicotinic receptors are involved in mediating morphine reward. Behav. Brain Res..

[67] Rezayof A., Nazari-Serenjeh F., Zarrindast M.R., Sepehri H., Delphi L. (2007). Morphine-induced place preference: Involvement of cholinergic receptors of the ventral tegmental area. Eur. J. Pharmacol..

[68] Dahan A., Kest B., Waxman A.R., Sarton E. (2008). Sex-specific responses to opiates: Animal and human studies. Anesth. Analg..

[69] Sarton E., Romberg R., Dahan A. (2003). Gender differences in morphine pharmacokinetics and dynamics. Adv. Exp. Med. Biol..

[70] Smulders C.J., Zwart R., Bermudez I., van Kleef R.G., Groot-Kormelink P.J., Vijverberg H.P. (2005). Cholinergic drugs potentiate human nicotinic alpha4beta2 acetylcholine receptors by a competitive mechanism. Eur. J. Pharmacol..

[71] Imhoff V., Rossignol B. (1983). Comparative effect of atropine on the adrenergic and muscarinic stimulation of phospholipid 32P labelling in isolated parotid cells: Atropine, a possible blocker of alpha-adrenergic receptors. Biol. Cell.

[72] Perera R.K., Fischer T.H., Wagner M., Dewenter M., Vettel C., Bork N.I., Maier L.S., Conti M., Wess J., El-Armouche A. (2017). Atropine augments cardiac contractility by inhibiting cAMP-specific phosphodiesterase type 4. Sci. Rep..

[73] Lomask M. (2006). Further exploration of the Penh parameter. Exp. Toxicol. Pathol..

