# Supplementary material for: L-Cysteine Ethyl Ester May Overcome Morphine-Induced Respiratory Depression by Activating Muscarinic Receptors
Source: Pharmaceuticals (Basel). 2026 Jul 21;19(7):1125. doi: 10.3390/ph19071125 (PMC13415194; doi:10.3390/ph19071125)
Supplement: Supplementary file 1 [file pharmaceuticals-19-01125-s001.zip › pharmaceuticals-4327922-supplementary.pdf]

Table S1

Definition of ventilatory parameters described in this study

Parameter	Abbreviation	Units	Definition
**A. Directly recorded parameters**			
Frequency of breaths	Freq	breaths/min	Rate of breathing
Inspiratory Time	Ti	sec	Duration of inspiration
Expiratory Time	Te	sec	Duration of expiration
End Inspiratory Pause	EIP	msec	Pause between end of inspiration and start of expiration
End Expiratory Pause	EEP	msec	Pause between end of expiration and start of inspiration
Relaxation time	RT	sec	Decay of expiration to 36% maximum
Tidal Volume	TV	ml	Volume of inspired air per breath
Peak Inspiratory Flow	PIF	ml/sec	Maximum inspiratory flow
Peak Expiratory Flow	PEF	ml/sec	Maximum expiratory flow
Expiratory flow at 50%	EF_{50}	ml/sec	Expiratory flow at 50% expired TV
Non-eupneic breathing index	NEBI	%	% of non-eupneic breaths per epoch
**B. Derived parameters**			
Minute Ventilation	MV = Freq x TV	ml/min	Total volume of air inspired per min
Ti/Te	Ti/Te	none	Inspiratory quotient
PEF/PIF	PEF/PIF	none	Flow balance
Expiratory Delay	Te-RT	No units	Difference in lengths of Te and RT
Inspiratory Drive	TV/Ti	ml/sec	Central urge to inhale
Expiratory Drive	TV/Te	ml/sec	Central drive to exhale
NEBI/Frequency	NEBI/Freq	%/(b/min)	Balanced rejection index

Figure S1

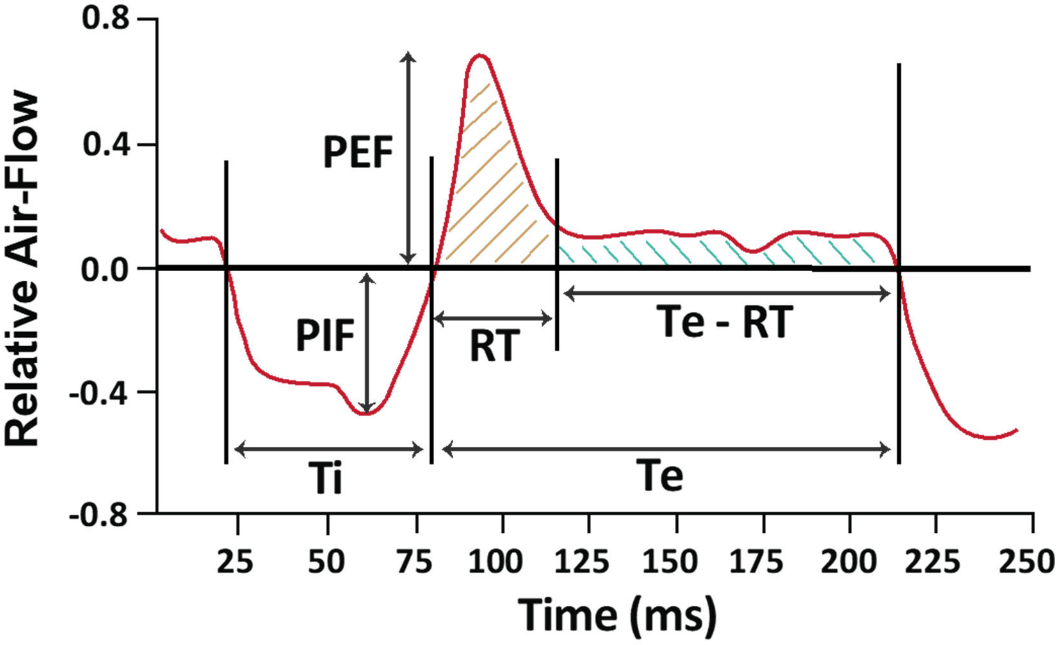

Supplemental Figure S1. Relationships between peak inspiratory flow (PIF), peak expiratory flow (PEF), relaxation time (RT) and expiratory time (Te).

Table S2

Baseline values in the two groups of rats used for the ventilatory studies

	Treatment Group	
**Parameter**	**Vehicle**	**Atropine**
N	6	6
Age, days	87.5 ± 0.6	87.1 ± 0.8
Body Weight, grams	363 ± 3	361 ± 3
Frequency of breathing (Freq), breaths/min	85.5 ± 2.6	86.4 ± 3.8
Tidal Volume (TV), ml	3.02 ± 0.08	3.05 ± 0.08
Minute Ventilation, ml/min	258 ± 11	263 ± 13
Inspiratory Time (Ti), sec	0.258 ± 0.010	0.254 ± 0.017
Expiratory Time, sec	0.523 ± 0.029	0.514 ± 0.034
Inspiratory Time/Expiratory Time	0.50 ± 0.02	0.51 ± 0.03
End Inspiratory Pause, msec	8.3 ± 0.2	9.1 ± 0.4
End Expiratory Pause, msec	32.3 ± 1.3	31.5 ± 2.0
Peak Inspiratory Flow (PIF), ml/sec	17.4 ± 0.3	17.5 ± 0.5
Peak Expiratory Flow (PEF), ml/sec	11.3 ± 0.3	11.7 ± 0.6
PIF/PEF	1.54 ± 0.04	1.52 ± 0.09
Rpef	0.14± 0.01	0.15 ± 0.02
$EF_{50,}$ ml/sec	0.46 ± 0.02	0.48 ± 0.03
Relaxation Time (RT), sec	0.30 ± 0.02	0.31 ± 0.04
Expiratory Delay (Te-RT)	0.22 ± 0.02	0.21 ± 0.01
Apneic Pause [Te/RT)-1]	0.77 ± 0.08	0.73 ± 0.04
Inspiratory Drive (TV/Ti), ml/sec	10.8 ± 0.5	12.4 ± 0.9
Expiratory Drive (TV/Te), ml/sec	4.9 ± 0.4	6.1 ± 0.3
Non-Eupneic Breathing Index (NEBI), % of epoch	2.9 ± 0.1	3.5 ± 0.2
NEBI/Freq, %/(breaths/min)	3.4 ± 0.2	4.1 ± 0.2

There were no between-group differences for any parameter (P >0.05, for all comparisons)

Table S3

Ages and body weights of the four groups of rats used for the righting-reflex studies

**Treatment groups**	**Number**	**Ages (days)**	**Body Weights (g)**
Vehicle + morphine + vehicle	9	88.3 ± 0.5	364 ± 2
Atropine + morphine + vehicle	9	88.0 ± 0.8	364 ± 3
Vehicle + morphine + L-CYSee	9	88.4 ± 0.6	362 ± 2
Atropine + morphine + L-CYSee	9	88.3 ± 0.9	363 ± 3

There were no between-group differences for any parameter ($P > 0.05$, for all comparisons)

Table S4

Comparison of the effects of atropine in naïve and morphine-treated rats

Parameter	Treatment Group	
	**Naïve***	**Morphine-treated**
Frequency (Freq), breaths/min	↑↑↑↑	No effect
Tidal Volume (TV), ml	↓↓	No effect
Minute Ventilation, ml/min	↑↑	No effect
Inspiratory Time (Ti), sec	↓↓↓	No effect
Expiratory Time, sec	↓↓↓↓	No effect
Inspiratory Time/Expiratory Time	↑	No effect
End Inspiratory Pause, msec	↑	↓↓↓
End Expiratory Pause, msec	↓↓↓	No effect
Peak Inspiratory Flow (PIF), ml/sec	↑↑↑	No effect
Peak Expiratory Flow (PEF), ml/sec	↑↑↑	No effect
PIF/PEF	No effect	No effect
Rate of achieving PEF (Rpef)	↑↑↑↑	↓↓↓
EF_{50}, ml/sec	↑↑↑↑	↓
Relaxation Time (RT), sec	↓↓↓	No effect
Expiratory Delay (Te-RT)	↓↓↓	No effect
Apneic Pause [Te/RT)-1]	↓↓↓	No effect
Inspiratory Drive (TV/Ti), ml/sec	↑↑	No effect
Expiratory Drive (TV/Te), ml/sec	↑↑	No effect
Non-Eupneic Breathing Index (NEBI), % of epoch	↑↑↑↑	No effect
NEBI/Freq, %/(breaths/min)	↑↑	No effect

*Based on data from: Getsy PM, May WJ, Young AP, Baby SM, Coffee GA, Bates JN, Lewis SJ., 2025b. L-cysteine ethyl ester activates muscarinic receptor signaling processes to overcome morphine induced respiratory depression in freely-moving rats. Neuropharmacology, in press. ↑, stimulatory effect. ↓, inhibitory effect. The number of arrows is based on the %change in the raw data.
